# Topographical Choroidal Thickness Change Following PDT for CSC: An OCT Case Report

**DOI:** 10.1155/2012/347206

**Published:** 2012-01-12

**Authors:** William J. Wirostko, Rick N. Nordgren, Adam M. Dubis

**Affiliations:** ^1^Department of Ophthalmology, The Eye Institute, Medical College of Wisconsin, 925 North 87th Street, Milwaukee, WI 53226, USA; ^2^Department of Cell Biology Neurobiology and Anatomy, Medical College of Wisconsin, 8700 West Wisconsin Avenue, Milwaukee, WI 53226, USA

## Abstract

*Purpose*. To describe topographical changes in choroidal thickness as measured by optical coherence tomography following photodynamic therapy (PDT) for central serous chorioretinopathy (CSC). *Methods*. Case report. *Results*. By 1 month following PDT, mean (SD) choroidal thickness decreased from 562 microns (24) to 424 microns (27) (*P* < 0.01) at 3 mm temporal to fovea, 483 microns (9) to 341 microns (21) (*P* < 0.01) at 1.5 mm temporal to fovea, 576 microns (52) to 370 microns (81) (*P* < 0.01) under the fovea, 442 microns (30) to 331 microns (54) (*P* < 0.04) at 1.5 mm nasal to fovea, and 274 microns (39) to 171 microns (17) (*P* < 0.01) at 3 mm nasal to fovea. The Location of greatest choroidal thickness (648 microns) prior to treatment was at point of leakage on fluorescein angiogram (FA). This region decreased to 504 microns following treatment. *Conclusion*. A decrease in choroidal thickness can be seen following PDT for CSC as far as 3 mm temporal and 3 mm nasal to fovea. The Location of greatest choroidal thickness may be at point of leakage on FA.

## 1. Introduction

Central serous chorioretinopathy (CSC) is characterized by a neurosensory retinal detachment in the posterior fundus associated with one or more leaks at the level of the retinal pigment epithelium (RPE) [1]. Etiology of leakage is unclear but thought to be related to hyperpermeability of the choroidal vasculature. Although many cases of CSC resolve spontaneously with improvement of vision, treatment may be considered for eyes with persistent or progressive vision loss from serous retinal detachment. Current treatment options include thermal laser photocoagulation or photodynamic therapy (PDT) with verteporfin (QLT Ophthalmics, Inc. Menlo Park, CA, USA) [1, 2].

Optical coherence tomography (OCT) is an imaging modality capable of depicting the retinochoroidal layers and the presence of a neurosensory retinal detachment in eyes with CSC [3]. In a recent OCT study, subfoveal choroidal thickness was shown to decrease following PDT for CSC [4]. However, the topographical location and extent of choroidal thickness changes following PDT for CSC were not described. In this paper, we describe a case report of topographic choroidal changes as measured by raster lines on spectral domain OCT following PDT for CSC. Findings are also described in relation to fluorescein angiography (FA) findings. 

## 2. Material and Methods

A retrospective case study of a patient receiving PDT with Visudyne for CSC was performed. Medical records were reviewed for clinical findings, FA results, OCT findings, and PDT treatment parameters. Outcome parameters included visual acuity, clinical findings, and choroidal thickness as measured by OCT images. Choroidal thickness measurements were obtained by exporting all OCT images into Image J (http://rsb.info.nih.gov/ij/) where number of pixels in choroidal layer was counted and converted into microns using micron/pixel ratio. All measurements were performed by one grader (RNN). The authors are not aware of any automated method to measure topographical choroidal thickness. Statistical analysis using the Mann-Whitney test was used to compare choroidal thickness before and after treatment at 3 mm temporal to the fovea, 1.5 mm temporal to the fovea, under the fovea, 1.5 mm nasal to the fovea, and 3 mm nasal to the fovea. Data set for each of these five locations was obtained from 5 horizontal raster scans (Figure 3).

## 3. Results

**Figure 1:**
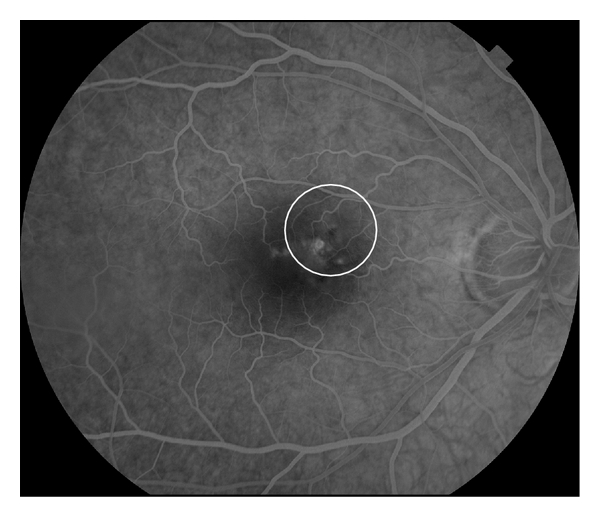
Fluorescein angiography of right eye demonstrating hyperfluorescence and leakage at level of RPE. White circle depicts the area of fundus treated with PDT (1.5 mm laser spot).

**Figure 2:**
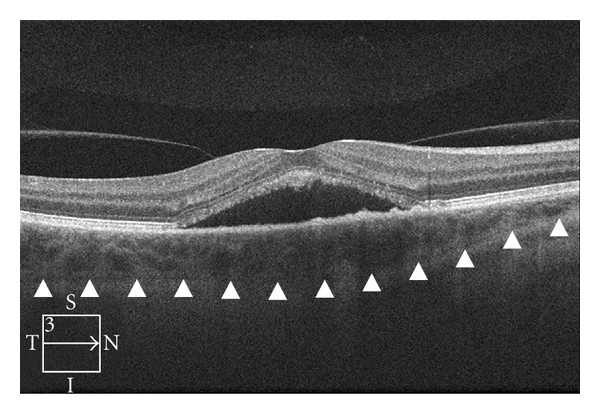
OCT image of retina and choroid prior to treatment demonstrating neurosensory retinal detachment. Arrowheads mark outer boundary of choroid used to measure choroidal thickness.

**Figure 3:**
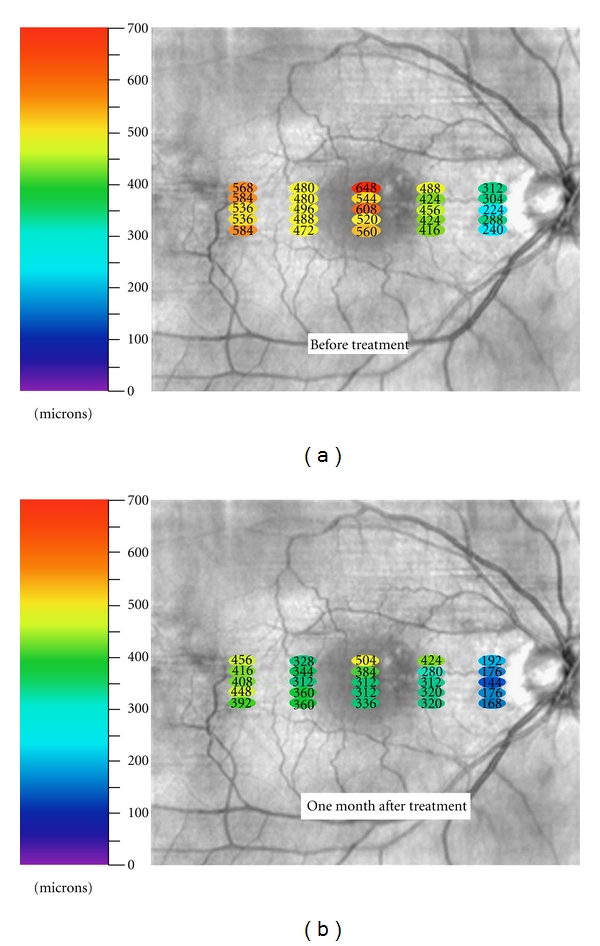
Fundus photograph of OD demonstrating thickness of choroid in microns at each specific measured point both before and after treatment.

A 52-year-old woman presented for decreased vision in her right eye of 1-month duration. Visual acuity was 20/60 OD and 20/20 OS. Fundus exam of OD revealed a neurosensory detachment of the fovea with abnormalities of the retinal pigment epithelium (RPE) just superonasal to the fovea. Fluorescein angiography depicted leakage superonasal to the fovea at the level of the RPE with pooling into the neurosensory space (Figure 1). Spectral domain OCT using Cirrus HD-OCT (Carl Zeiss Meditec, Inc., Dublin, CA, JUSA) demonstrated a neurosensory detachment of the fovea (Figure 2). Five 6 mm long horizontal raster lines centered on the fovea and spaced 0.25 mm apart were obtained to topographically map the choroidal thickness. Mean (SD) choroidal thickness was 562 microns (24) at 3 mm temporal to fovea, 483 microns (9) at 1.5 mm temporal to fovea, 576 microns (52) under the fovea, 442 microns (30) at 1.5 mm nasal to fovea, and 274 microns (39) at 3 mm nasal to fovea. Thickest area of choroid (648 microns) was under area of leakage as seen on FA (Figure 3). Diagnosis of CSC was established, and treatment with PDT was recommended. After discussing the off-label nature of PDT for CSC, patient chose to proceed with treatment. Photodynamic therapy with verteporfin using 1.5 mm laser spot size, standard treatment parameters, and 83 seconds of duration was applied to juxtafoveal area of leakage as guided by FA [5]. Care was taken to avoid directly treating the fovea (Figure 1).

**Figure 4:**
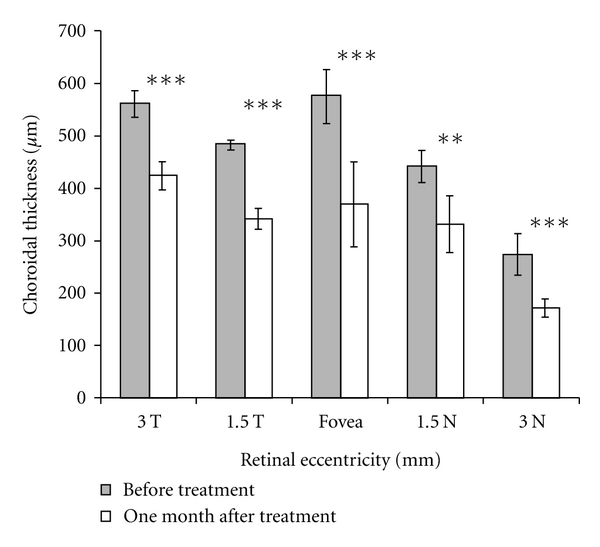
Chart comparing thickness of choroid at 3 mm temporal to fovea, 1.5 mm temporal to fovea, under the fovea, 1.5 mm nasal to fovea, and 3 mm nasal to fovea before and after treatment with PDT. Error bars represent one standard deviation. At all points, there was a significant reduction in choroidal thickness (the Mann-Whitney test): ***P < 0.01, **P < 0.04.

 At 1 month following treatment, visual acuity improved to 20/20. Optical coherence tomography demonstrated resolution of subretinal fluid. Mean (SD) posttreatment choroidal thickness measurements were 424 microns (27) at 3 mm temporal to fovea, 341 microns (21) at 1.5 mm temporal to fovea, 370 microns (81) under the fovea, 331 microns (54) at 1.5 mm nasal to fovea, and 171 microns (17) at 3 mm nasal to fovea (Figure 3). This reduction was statistically significant (*P* < 0.01, *P* < 0.01, *P* < 0.01, *P* < 0.04, *P* < 0.01) at each location, respectively, using the Mann-Whitney test (Figure 4). Point of prior greatest choroidal thickness (648 microns) decreased to 504 microns, but was still the point of greatest choroidal thickness (Figure 3).

## 4. Discussion

This study describes topographical thickness changes in the choroidal layer using OCT for an eye with CSC undergoing PDT with verteporfin. Thickness changes are described with regard to FA findings and treatment location. The authors are not aware that this has been previously described. Prior reports on choroidal thickness in CSC have only described subfoveal thickness findings with no reference to location of FA leakage or PDT laser spot. Understanding the topographic changes of the choroid following PDT for CSC may be important, both for advancing our understanding of CSC and also improving our ability to restore vision. 

 Our findings of decreased choroidal thickness following PDT concur with prior reports [4]. In 2010, Maruko et al. found subfoveal choroidal thickness decreased from 389 ± 106 micron at baseline to 330 ± 103 microns at one month [4]. Our patient's mean (SD) subfoveal choroidal thickness decreased from 576 microns (52) to 370 microns (81) (*P* < 0.01 Mann-Whitney test). Interestingly, our report suggests that the reduction in choroidal thickness is diffuse and extends further than just under the fovea as previously reported. We measured a statistically significant reduction in choroidal thickness from up to 3 mm temporal of the fovea to 3 mm nasal of the fovea following a 1.5 mm juxtafoveal PDT laser spot applied to an area of juxtafoveal leakage as seen on FA (Figure 4). It remains unclear whether a similar diffuse effect or as significant of an effect would occur if the PDT laser spot was not applied over the leakage as seen on FA. Distinguishing these effects may be important for some cases, especially when FA leakage is subfoveal and the treating physician wishes to avoid exposing the fovea to PDT laser.

It is interesting to observe that the single thickest measurement of the choroid (648 microns) before treatment was in the area of leakage as seen on FA. Following treatment, this single location decreased to 504 microns but was still the point of greatest choroidal thickness (Figure 3). Unfortunately, the authors cannot comment on how abnormal either of these measurements is since complete normative data on choroidal thickness correcting for age, race, refractive area, and fundus location is not available [6].

 Limitations of our study include the single sample size, the retrospective nature, short duration of follow-up, and limited sampling of choroid. Additionally, indocyanine green angiography which can provide information on choroidal hyperpermeability was not obtained on this patient. Nonetheless, the authors feel publishing this case is valuable since its unique combination of small focal juxtafoveal leakage on FA and small PDT laser spot allows us to make some interesting observations. Certainly, further studies are needed to corroborate our findings.

## 5. Conclusion

This study documents the topographical changes in the choroidal layer as measured with OCT in an eye undergoing treatment with PDT and verteporfin for CSC. A reduction in choroidal thickness was observed as far away as 3 mm temporal and nasal to the area of leakage as seen on FA following treatment. Point of greatest choroidal thickness before and after treatment was in the area of leakage on FA.
